# A Case of Circumscribed Choroidal Hemangioma Treated With Proton Beam Therapy and Followed Up for 15 Years

**DOI:** 10.7759/cureus.52389

**Published:** 2024-01-16

**Authors:** Daichi Takizawa, Toshiyuki Okumura, Masashi Mizumoto, Kei Nakai, Hideyuki Sakurai

**Affiliations:** 1 Radiation Oncology, Hitachi General Hospital, Hitachi, JPN; 2 Radiation Oncology, Ibaraki Prefectural Central Hospital, Kasama, JPN; 3 Radiation Oncology, University of Tsukuba, Tsukuba, JPN

**Keywords:** benign tumors, retinal detachment (rd), long term follow up, choroidal haemangioma, proton beam radiotherapy

## Abstract

Circumscribed choroidal hemangiomas are rare and benign tumors but often have a progressive course and are complicated by retinal detachment and glaucoma. The effectiveness of external radiation for large tumors that are difficult to treat with photodynamic therapy was recently reported; however, few studies have conducted long-term follow-ups. We encountered a case of localized choroidal hemangioma that was treated with proton beam therapy and followed up for 15 years. A 37-year-old man was diagnosed with a 10 × 4 mm circumscribed choroidal hemangioma involving the macular area with retinal detachment. Proton beam therapy was performed at 26.4 Gy relative biological effectiveness (RBE) in 8 fractions. The choroidal hemangioma gradually shrank over three years, and the retinal detachment also improved. A cataract developed on the affected side 11 years after irradiation, and eye coordination issues developed 15 years after irradiation. Glaucoma was not observed during the follow-up period; however, visual acuity did not recover, and the patient developed light perception. Although vision was not preserved, proton beam therapy effectively shrank the tumor and maintained quality of life.

## Introduction

Uveal hemangioma is a rare disease that mainly affects the posterior pole of the choroid but may also occur in the iris and ciliary body [1]. It is divided into two subtypes: circumscribed and diffuse. The diffuse type is often associated with Sturge-weber syndrome. Pathologically, it is a benign vascular hamartoma. It often occurs unilaterally, and symptoms include decreased visual acuity and hyperopia due to serous retinal detachment and macular edema associated with the tumor, as well as secondary fibrosis or atrophy of the retinal pigment epithelium [2]. Diffuse cases frequently develop glaucoma as a complication [3].

Since uveal hemangioma is a benign tumor, observations are an option if there are no symptoms. Treatment is aimed at preventing complications and vision loss. Photodynamic therapy is often the first choice, and other treatments include transpupillary thermotherapy and vitrectomy [4]. Large or diffuse hemangioma may involve the optic nerve within the treatment area; therefore, optic neuritis is risky, making it difficult to treat with photodynamic therapy alone. Additionally, large hemangiomas often require multiple sessions of photodynamic therapy, which can increase the risk of choroidal atrophy and neurosensory retinal degeneration [5]. The effectiveness of treatment with external radiation was recently reported, particularly for large hemangiomas and hemangiomas involving the optic disc [6]. However, most cases treated with external beam radiation therapy have been reported since the late 1990s, and few studies have performed long-term follow-ups [3,7]. We herein report a case of large focal choroidal hemangioma with severe retinal detachment, including the macular area, which was treated with proton beam therapy and followed up for 15 years.

## Case presentation

The patient was a 37-year-old man with visual impairment and unremarkable medical and family histories. Four years before the proton beam therapy, vision in the left eye declined; however, he did not wish to undergo further investigation. One year before the proton beam therapy, the left eye began to spontaneously turn inwards. The patient visited a nearby ophthalmology clinic; his visual acuity was hand motion, and a left choroidal tumor was detected, and he was referred to our hospital.

**Figure 1:**
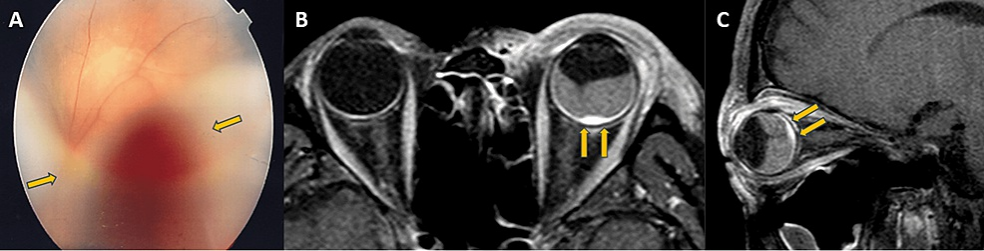
Color fundus photography (A) and contrast-enhanced MRI (B, C) of circumscribed choroidal hemangioma before proton beam therapy. Color fundus photography (A) showed retinal detachment from the optic disc to the macula  (arrows in A). Contrast-enhanced MRI (B, C) showed extensive retinal detachment and 10×4 mm hemangioma in the left eye (arrows in B, C).

During his first visit to the Ophthalmology Department, visual acuity was hand motion. Clinical examination revealed central serous retinal detachment, and there could be some signs of the reddish color of the hemangioma, estimated to be 6 disc diameters under the detachment (Figure 1A). In addition, MRI revealed extensive retinal detachment in the left eye and 10 × 4 mm hemangioma with strong contrast enhancement with gadolinium at the base of the eyeball (Figure 1B, 1C). After further B-scan ultrasound, CT scan, and physical examination, the patient was diagnosed with a circumscribed choroidal hemangioma. The tumor involved the optic nerve, and it was thought that it would be difficult to treat with photodynamic therapy alone. After receiving informed consent regarding treatment options from an ophthalmologist, the patient requested proton beam therapy.

**Figure 2:**
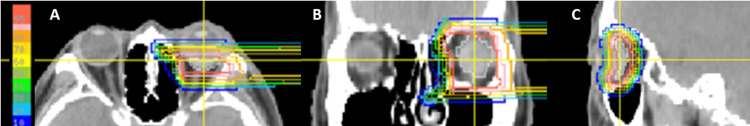
Dose distribution map of proton beam therapy for circumscribed choroidal hemangioma The images show axial (A), coronal (B), and sagittal (C) CT images. The clinical target volume was the hemangioma, and a setup margin of 2 mm was added to create a planning target volume.

He was referred to our department for proton beam therapy. A titanium ring for proton beam aiming was sewn onto the sclera of the left eye near the lesion, and proton beam therapy was started. Referring to previous proton beam therapy reports, the total dose irradiated was 26.4 Gy relative biological effectiveness (RBE) in 8 fractions over 14 days [8]. During irradiation, the eyes were opened to keep the lens in the front position, and irradiation was performed from the left side, avoiding the lens and using a single portal. The dose distribution map of proton beam therapy showed the dose to the right eye to almost zero (Figure 2).

**Figure 3:**
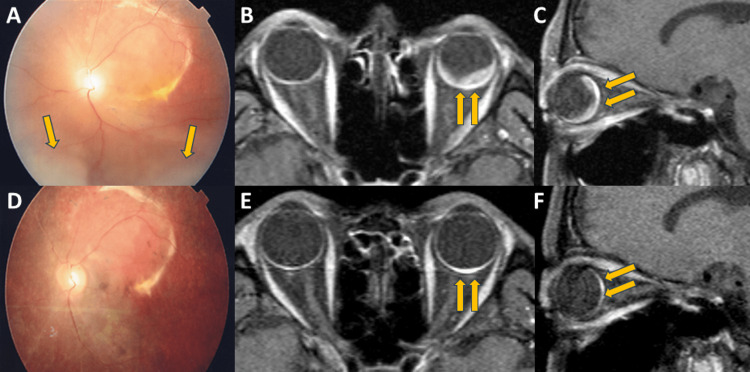
Color fundus photography (A, D) and contrast-enhanced MRI (B, C, E, F) of circumscribed choroidal hemangioma after proton beam therapy (A-C) One year after proton beam therapy. (D-F); Three years after proton beam therapy. (A, D) Color fundus photography revealed that the retinal detachment had improved one year after proton beam therapy, but some remained (arrows in A). It disappeared three years after proton beam therapy. (B, C, E, F) Contrast-enhanced MRI revealed gradual shrinkage of hemangioma over three years; however, a small lesion with contrast enhancement persisted (arrows in B, C, E, F).

As a result of color fundus photography, the retinal detachment in the left eye improved 1 year after proton beam therapy, and the retinal detachment disappeared 3 years later (Figure 3A, 3D). In addition, MRI showed gradual shrinkage of hemangioma over 3 years; however, a small lesion with contrast enhancement persisted but remained unchanged thereafter (Figure 3B-3C, 3E-3F).

Visual acuity did not recover, and the patient continued to have light perception. Although Schirmer’s test showed a difference in lacrimal secretions between the left and right sides, there was no subjective difference. Eleven years after irradiation, he was diagnosed with a mild cataract due to clouding of his left lens. Moreover, he did not need to undergo surgery. Thirteen years after irradiation, the patient developed a sunken feeling in his left eye, and the examination revealed enophthalmos and orbital fat atrophy. Fifteen years after irradiation, an abduction deficit of the left eye was observed, and a coordination disorder developed when looking to the left. Coordination was possible in other directions. Glaucoma was not detected in 15 years of follow-ups. Furthermore, no complications were observed in the right eyeball.

## Discussion

Pathologically, the progressive expansion of focal choroidal hemangioma is attributed more to venous congestion than the growth of the tumor itself [9]. The effects of radiation therapy are generally explained by vascular occlusion due to thrombus formation and vascular fibrosis within tumor blood vessels and the effects of suppressing the proliferation of vascular endothelial cells within the tumor [8]. Recurrence after photodynamic therapy, the first-line treatment, has been reported in approximately 40% of patients. Repeated retinal photocoagulation is required, resulting in poor visual acuity of less than 0.1 on average [10]. External beam radiation therapy is highly effective, and irradiation with approximately 20 to 25 Gy reduced tumor sizes and improved retinal detachment in more than 80% of cases [7].

Regarding the general dose fractionation of radiation therapy, the literature reports that the prescription dose is approximately 18Gy to 30Gy in 4 to 10 fractions [6-8]. External beam radiation therapy has the advantage of treating large hemangiomas involving the optic nerve compared to photodynamic therapy and transpupillary thermotherapy but has disadvantages such as slow absorption of subretinal fluid and cost [2]. In addition, high cost and limited availability are major drawbacks of proton beam therapy. The side effects of radiation therapy include cataracts, radiation optic neuropathy, and radiation maculopathy [5,7].

Regarding the selection of proton beam therapy and X-ray therapy, proton beam therapy has the advantage of a steep Bragg peak and delivering a lower radiation dose to normal tissue surrounding the tumor [11]. The therapeutic effects of X-ray therapy and proton beam therapy for choroidal hemangiomas do not significantly differ [12]; however, even at doses of 20 Gy or less, which are used for benign diseases, the risk of radiation-induced cancer increases over time [13]. Therefore, in long-term follow-ups, proton beam therapy may be more advantageous regarding side effects, such as secondary carcinogenesis.

In the present case, the tumor was sufficiently large to reach the macula at the time of the initial diagnosis; therefore, it was not possible to preserve vision. However, the tumor was reduced by proton beam therapy, the course progressed without the complication of glaucoma, and the quality of life improved. In some untreated choroidal hemangiomas, progressive subretinal fluid is known to cause neovascular glaucoma [5]. However, we found no case reports of glaucoma, including radiation therapy-related secondary glaucoma, after proton beam therapy for circumscribed choroidal hemangioma. Cataracts are known to be the most frequent side effect of proton beam therapy for choroidal hemangioma [7]. Although the irradiation field avoids the lens, an increase in the exposure dose of only 10 mSV has been associated with a higher risk of lens opacity [14]. Miguel et al. reported that after radiotherapy for 243 eyes with uveal melanoma, approximately one-third of patients suffered from cataracts at 3 years, progressively increasing to 60% at 15 years [15]. Radiation-induced cataracts may be detected more frequently with long-term follow-up.

Additionally, it has been reported that the lower the exposure dose to the lens, the more likely the cataract will be mild [16]. There is also a report that most cataracts after proton beam therapy for choroidal hemangioma, which was irradiated avoiding lens, were mild and did not require treatment [7]. That also applies to this case-a cataract and coordination disorder developed in the left eye more than 10 years after treatment. Since the patient’s right eye progressed without any symptoms, they were considered to be late side effects of radiation therapy. To our knowledge, this is the first case report of choroidal hemangioma being followed up for 15 years after radiotherapy. We intend to accumulate more cases in the future.

## Conclusions

We encountered a case of circumscribed choroidal hemangioma treated with proton beam therapy and followed up for 15 years, more than 10 years after radiation therapy, a cataract and loss of coordination developed in the affected eye only. Although vision was not saved, proton beam therapy effectively shrank the tumor and maintained quality of life.

## References

[1] Witschel H, Font RL (1976). Hemangioma of the choroid. a clinicopathologic study of 71 cases and a review of the literature. Surv Ophthalmol.

[2] Sen M, Honavar SG (2019). Circumscribed choroidal hemangioma: an overview of clinical manifestation, diagnosis and management. Indian J Ophthalmol.

[3] Schilling H, Sauerwein W, Lommatzsch A, Friedrichs W, Brylak S, Bornfeld N, Wessing A (1997). Long-term results after low dose ocular irradiation for choroidal haemangiomas. Br J Ophthalmol.

[4] Shukla D, Ramasamy K (2007). Vitrectomy for circumscribed choroidal hemangioma with exudative retinal detachment refractory to transpupillary thermotherapy. Indian J Ophthalmol.

[5] Karimi S, Nourinia R, Mashayekhi A (2015). Circumscribed choroidal hemangioma. J Ophthalmic Vis Res.

[6] Ritland JS, Eide N, Tausjø J (2001). External beam irradiation therapy for choroidal haemangiomas. visual and anatomical results after a dose of 20 to 25 Gy. Acta Ophthalmol Scand.

[7] Levy-Gabriel C, Rouic LL, Plancher C (2009). Long-term results of low-dose proton beam therapy for circumscribed choroidal hemangiomas. Retina.

[8] Hannouche D, Frau E, Desjardins L, Cassoux N, Habrand J, Offret H (1997). Efficacy of proton therapy in circumscribed choroidal hemangiomas associated with serious retinal detachment. Ophthalmology.

[9] Shields JA, Stephens RF, Eagle RC Jr, Shields CL, De Potter P (1992). Progressive enlargement of a circumscribed choroidal hemangioma. a clinicopathologic correlation. Arch Ophthalmol.

[10] Anand R, Augsburger JJ, Shields JA (1989). Circumscribed choroidal hemangiomas. Arch Ophthalmol.

[11] Mizumoto M, Murayama S, Akimoto T (2016). Proton beam therapy for pediatric malignancies: a retrospective observational multicenter study in Japan. Cancer Med.

[12] Höcht S, Wachtlin J, Bechrakis NE (2006). Proton or photon irradiation for hemangiomas of the choroid? a retrospective comparison. Int J Radiat Oncol Biol Phys.

[13] McKeown SR, Hatfield P, Prestwich RJ, Shaffer RE, Taylor RE (2015). Radiotherapy for benign disease; assessing the risk of radiation-induced cancer following exposure to intermediate dose radiation. Br J Radiol.

[14] Mrena S, Kivelä T, Kurttio P, Auvinen A (2011). Lens opacities among physicians occupationally exposed to ionizing radiation--a pilot study in Finland. Scand J Work Environ Health.

[15] Miguel D, de Frutos-Baraja JM, López-Lara F, Saornil MA, García-Álvarez C, Alonso P, Diezhandino P (2018). Radiobiological doses, tumor, and treatment features influence on outcomes after epiescleral brachytherapy. a 20-year retrospective analysis from a single-institution: part II. J Contemp Brachytherapy.

[16] Banou L, Tsani Z, Arvanitogiannis K, Pavlaki M, Dastiridou A, Androudi S (2023). Radiotherapy in uveal melanoma: a review of ocular complications. Curr Oncol.

